# Science, safety, and trust: the case of transgenic food

**DOI:** 10.3325/cmj.2013.54.91

**Published:** 2013-02

**Authors:** Lucia Martinelli, Małgorzata Karbarz, Helena Siipi

**Affiliations:** 1 Museo delle Scienze, Trento, Italy; 2 University of Rzeszów, Institute of Applied Biotechnology and Basic Sciences, Kolbuszowa, Poland; 3 University of Turku, Department of Behavioural Sciences and Philosophy, Turku, Finland

## Abstract

Genetically modified (GM) food is discussed as an example of the controversial relation between the intrinsic uncertainty of the scientific approach and the demand of citizen-consumers to use products of science innovation that are known to be safe. On the whole, peer-reviewed studies on GM food safety do not note significant health risks, with a few exceptions, like the most renowned “Pusztai affair” and the recent “Seralini case.” These latter studies have been disregarded by the scientific community, based on incorrect experimental designs and statistic analysis. Such contradictory results show the complexity of risk evaluation, and raise concerns in the citizen-consumers against the GM food. A thoughtful consideration by scientific community and decision makers of the moral values that are present in risk evaluation and risk management should be the most trustable answer to citizen-consumers to their claim for clear and definitive answers concerning safety/un-safety of GM food.

In this essay in the series of articles from “Bio-Objects” research network supported by the Cooperation in Science and Technology (COST) program (1), we focus on genetically modified (GM) plants for food production as a remarkable example of a biotechnology innovation fitting the “bio-object” classification. GM plants are defined as organisms whose genomes have been modified applying recombinant techniques (rDNA) by transferring extra genes or modulating (knockdown or knockout) genes already present in the species, with the aims of acquiring knowledge on gene functions, obtaining genetic improvement, and yielding selected compounds (2).

GM plant generation dates back already 30 years when at the Miami Winter Symposia of January 1983, three independent groups announced successful transfer of bacterial genes into plants, producing tobacco and petunia resistant to antibiotics (3-5). A few months there followed an insertion of a plant gene from one species into another species, generating a sunflower expressing the bean phaseolin gene (6). Thereafter, gene transfer technology increased dramatically while expectations on applications in agro-food genetic improvement were progressively rising. Besides overcoming conventional breeding constraints, solutions of crucial worldwide human questions were foreseen, such as adequacy of food resources to be available to the increasing world population and in particular to the hungry countries; generation of healthier food with enhanced nutritional values; development of an agricultural practice more respectful to environmental issues, based on crops constructed to be intrinsically resistant to the most relevant pests and diseases, thus free from chemical protection.

As extensively reported in literature, molecular tool applications in agriculture, human health, and food offer today remarkable opportunities even though more promises than concrete achievements on the market have been accomplished. The further achievements are continually expected based on the information accumulated through genetic research advancements (7).

## GM plants as “bio-objects”

From 2000, while use of GM plants in agriculture was increasingly becoming a consolidated practice and novel GM foods were entering into the market with a globally upward trend reaching nowadays 160 million hectares cultivated with biotech crops, concerns and passionate social and political controversies replaced enthusiastic expectations from the biotech era (7). Biotechnology application in agriculture soon became – and still is – a problematic issue and various countries all over the world gradually adopted own regulations for production, cultivation, import, and traceability of GM crops and their derivates to meet public demand of safety and to manage (perceived/true) technical risks, while biotech public research suffered from funding cut off.

This new course, which we regard as a significant step of a “bio-objectification” process, well portraits the controversial interactions occurring when science innovations break into society. GM plants, accordingly, as other “biological creatures” of agriculture and medicine research, bear some crucial features of “bio-objects.” They are constructed and manipulated biologies on the fine line between “natural” and “non-natural”/“artificial” that have hybridity (thus evoking the language of the “unnatural”) and are potentially useful for enhancing human life quality, resulting in the challenge of conventional natural, cultural, scientific and institutional orderings (8,9). Moreover, they have potential to move between domains, shifting from agriculture (the “first-generation GM plants,” whose modifications are aimed at solving agronomic constraints), nutrition (the “second-generation” GM plants, whose modifications are aimed at enhancing nutritional values) and health and industry (the “third generation” GM plants, whose modifications are aimed at farming specific compounds to be adopted in pharmaceutical and health care).

Bio-social impacts of GM plants have been extensively reported in literature (10) and at the Web sites of various associations and no-profit organizations involved in social issues and environment protection, while perceived risks related to hybridity and “crawling” across genetic barriers (11), as well as the significance of human intervention in Nature (12), have been already considered.

Here, focusing on human health risk, as evaluated by scientific community and institutional organs, we aim to discuss GM food as an example of “bio-object,” which enlightens the controversial relation between the intrinsic uncertainty of the scientific approach and the demand of citizen-consumers to use the products of science innovation that are known to be safe.

## Risk evaluation

Release of GM crops in open field and on the market is authorized all over the world according to various regulations and policies of different countries, and in Europe according to Reg. 1829/2003/EC. Moreover, “the European Union guarantees the traceability and labelling of GMOs and products produced from these organisms throughout the food chain. Traceability allows the monitoring and checking of information given on labels, the monitoring of effects on the environment and the withdrawal of products from the market in cases where new scientific data demonstrate that the GMOs used in the product present an environmental or health risk” (Reg. 1830/2003/EC). Within this regulatory framework, specific recommendations were formulated by EFSA (13). Accordingly, the evaluation of GM plants’ potential effects on the environment are based on a case-by-case basis, following a step-by-step assessment approach, which takes into account crucial aspects of hazards and risks such as their persistence, invasiveness, and interactions with other organisms, the production systems, the receiving environment, and the biogeochemical processes, as well as their effects on human and animal health. This evaluation is meant to be supported by independent experts and based on the most accredited and updated scientific knowledge on the topic.

After 1995, assessment of health impact of GM plants has been the subject of extensive peer reviewed scientific literature, which has been mostly focused on maize, soybean (the primary transgenic crops distributed on the market), rice, and potato. Together with in vitro analysis, long-term and multigenerational feeding studies were mainly performed on rats as model system, besides mice, cows, and fish, by assessing body and organ weight, hematological values, enzyme activities, organ and tissue histopathological examination and transgenic DNA detection. According to comprehensive studies (14), in which the most accredited scientific papers on feeding trials have been analyzed on the basis of certified experimental and statistical parameters (15,16), no significant health risks were found, and possible differences detected between transgenic feedings and their isogenic counterparts were considered of no biological or toxicological significance. Worth stressing, in the few studies where indications of no nutritional equivalence or altered parameters were reported, thus supporting health hazard, severe incorrect experimental designs with detrimental effects on statistical analysis have been advocated within the scientific community, hence rejecting these results (14).

## Controversial cases

Among the first animal feeding studies on GM diet to be independently peer reviewed, the most renowned is the one conducted at the Rowett Research Institute, Scotland, also known as “Pusztai affair” (17), which resulted for the researcher in suspension and banning from speaking publicly, and ended up with the not renewing his annual contract. Also co-author reported on suffering from mobbing, while *The Lancet,* which published this work as a letter was object of criticism. This study aimed at evaluating the effects of short-term rat feeding with GM potatoes expressing the lectin *Galanthus nivalis* agglutinin (GNA) gene developed to increase nematode and insect resistance. Histological observations of the stomach, jejunum, ileum, cecum, and colon showed that the presence of GNA in the diets, irrespective of whether originating from transgenic potatoes or from control potato diets supplemented with GNA, was associated with significantly greater mucosal thickness of the stomach when compared with controls. By contrast, a potent proliferative effect on the jejunum was observed in GM potato-based diet, an outcome not observed in controls or in rats fed with control potatoes but added with GNA. This latter result was interpreted as the effect of the gene transfer technique, such as the plant vector used for transferring the exogene or some form of positioning effect in the potato genome caused by the exogene insertion. Two official audits (respectively by Rowett Institute and the Royal Society) stated that the data did not support conclusions and severe experimental drawbacks were remarked, such as poorly designed experiments, presence of uncertainties in the composition of diets, inadequate rat number, incorrect statistical methods, and lacking consistency within experiments. On the other hand, this study has been the banner of anti-GMO movement for attributing interference by biotech companies on GM safety evaluation.

The “Seralini case” (18) is the most recent example of controversy associated with scientific publications on GM food evaluation. Authors aimed at assessing the long-term toxicity of the commercial formulation of Roundup herbicide and the maize line NK603 (Monsanto Corp., USA) harboring the gene encoding a glyphosate tolerant form of the enzyme 5-enolpyruvylshikimate-3-phosphate synthase (EPSPS) and developed to allow the use of the herbicide glyphosate as a weed control option in corn (19). As compared with its nearest isogenic non transgenic counterpart, rat feeding for two years with maize NK603 with or without supplements of the herbicide, resulted in severe kidney nephropathies and a significant sex-dependent increased mortality, development of large mammary tumors in females and liver congestions and necrosis in males. These outcomes were explained as a non linear endocrine-disrupting effects of herbicide as well as the overexpression of the transgene in the GM maize and its metabolic consequences. Together with the data originated from the study, doubts on the reliability of official risk evaluation methods were raised, in particular concerning duration of the long-term evaluation (15,16). Moreover, in the concluding remarks, further studies were forecasted concerning the assessment of “other mutagenic and metabolic effects of the edible GMO, which, according to Authors, ‘cannot be excluded’” (18).

These alarming results and related pictures of rats bearing tumors resonated in the media and on the internet, opening a renewed concern in citizen-consumers against the use of biotech applications in food and feed, and motivating criticism by various actors involved in biotech matters (20). As for governments, the French and Russian launched investigations into the safety of NK603, and Russia and Kazakhstan placed temporary bans on its imports. Scientific community – with few exceptions (21) – replied with a quantity of opinions and response letters from top scientists, where the Seralini study was dismissed and a more solid peer-review system in scientific journals was claimed for (22). As for institutions deputed to safety evaluation, EFSA delivered its final statement (also in agreement with the independent assessments by organizations of Belgium, Denmark, France, Germany, Italy, and the Netherlands) (23), which recommended rejection of this paper as scientifically unsound and stated a no need to re-examine its previous safety evaluations of maize NK603. Weaknesses in the methodology and experimental design, leading to misleading conclusions, were the basic faults assessed in this paper in particular deriving from the use of inappropriate animal line bearing a natural tumor formation rate of more than 50% and the minimal size of animal sample which was in contrast with the internationally recommended standards for a proper nutritional or toxicological assessment of a GM line. Controversial results concerning the dose-dependency between mortality or cancerogenesis of either the herbicide supplemented- or the maize NK603-diets were also pointed out.

## Science, safety, and trust

A proper scientific risk evaluation requires specific scientific knowledge, and, as above described, controversies regarding risk evaluation are still common within the scientific community. This makes lay people, in their safety considerations, dependent on interpretations and explanations provided by scientists and the media. Accordingly, the question of trust is inherently embedded in the safety discussion. Because of the progressive collection of data and uncertainties presented above, GM food may be regarded as a “bio-object” that crosses back and forward the boundaries of “safe/unsafe” and “well known/still to be known.” Thus, it is worth asking, how should the controversial relation between the intrinsic uncertainty of the science and citizen-consumers’ desire to eat food that is known to be safe be understood and managed?

It should be pointed out that citizen-consumers are quite well aware of uncertainty features of scientific knowledge and are not demanding or expecting a “zero risk:” they rather complain that uncertainties are not taken seriously enough in decision-making concerning GMOs and in risk communication with the public (24). This may be a part of the reason for their unwillingness to consume GM food, as long as no specific benefits from choosing GM products are perceived (25,26). Besides, the unwillingness to eat GM food cannot be explained merely by referring to consumers’ lack of knowledge regarding the risk evaluation. The deficit model type of thinking (the paradigm “more knowledge – more acceptance”) has been criticized on theoretical and empirical grounds for overemphasizing the role of scientific ignorance in attitude formation (11,27-29). Nonetheless, it should be remarked that this assumption is still a common mindset in the scientific community, and shapes science communication, public engagement initiatives, and policymaking (29-31). Thus, it has been suggested that scientists and decision-makers should concentrate in being trustworthy, instead of focusing merely on providing information about scientific and values issues (32). But how to be trustworthy?

## Moral values and value evaluations

Risk evaluation and risk management are usually presented as fundamentally and primarily scientific undertaking. In the “Pusztai affair” and “Seralini case,” for example, the public and academic discussion was related merely to scientific issues, or at least issues that have been presented as a matter of science. However, moral value questions –evaluations on what is morally right and wrong, desirable and undesirable – are necessarily present in risk evaluation and risk management. These include: How big risks are acceptable? Which risks should we take? How safe is safe enough? Which of the identified possible consequences are risks (undesirable) and which benefits (desirable)? How severe are the identified risks? To whom may the risks fall? Which are the suitable objects of comparison (33-35)? The aim of science (truth) and risk analysis (safety) are not the same, and risk analysis is intimately connected to the following question: Which should be a sufficient amount of evidence for safety or unsafety claims? In the “Pusztai affair” and “Seralini case,” the critics necessarily took a stand in this question when stating that these studies did not provide sufficient evidence for unsafety of a GM crop. We suggest that the controversy as well as the problem of trust may at least partly lie in a mistaken assumption that views concerning these moral value questions are commonly shared in the academia as well as in public sphere, as already pointed out: “what is typically called ‘public rejection of science’ is properly described as public rejection of commitments based on value commitment that are misunderstood and misrepresented by scientists and policy experts as solely scientifically determined” (36). Thus, building trust, as well as understanding and solving the controversy, requires making the moral values visible for all parties concerned and accepting them as topic of both public and academic discussion.

If we are right about the presence of value questions and disagreements concerning values in risk evaluation and management, being trustworthy may require acknowledging them and spelling them out in science communication. However, it has been noted that “being trustworthy cannot be limited to increasing transparency and providing information to consumers;” it further requires acting in a predictable manner, taking one’s responsibilities seriously (32), and maybe also *“*including citizen-consumers into decision-making” (37). The requirement for engaging the public in the decision-making concerning GM plants is also pointed out by European Union (Reg. 2001/18/EC), according to which *“member states shall […] consult the public and, where appropriate, groups on the proposed deliberate release*.” The European practice, however, has been criticized as being too concentrated on purely scientific points and less concerned about the value questions, which seem to be left without notice. Since most citizen-consumers are unable to carry out scientific risks evaluations, the consultation practice leaves them a very limited (if not absent) possibility to really affect the decisions made (38). Thus, if building trustworthiness requires real (not just apparent) possibilities to affect decision, the current European practice seems unlikely to contribute to being trustworthy.

## Uncertainty and demand for safety

Finally, we would like to ask whether the question “Are GM crops safe/dangerous to human health?” is sensible and should it be the topic of public discussion. It is certainly true that GM techniques could be used to develop plants that are dangerous to human health (for example poisonous variants of common crop plants). That possibility, however, does not imply that the way the GM technique is used today is likely to lead into dangerous outcomes. Thus, the question intended in a literal form is left without a definitive answer, as science innovations are on the same time “results of science knowledge” and “carriers of new questions to be investigated.” This question, therefore, may even be considered too broad and thus unanswerable. For these reasons, giving a simple yes/no answer to the query concerning safety of GMOs is impossible. Rather, we should concentrate on more definite answerable questions and in so doing emphasize the “case-by-case” evaluation of GM plants, where each individual product of biotech innovation – instead of the technique in its whole – is thoroughly assessed.

In conclusion, the most suitable answer to the “big question” raised by the consumers, “Can science give clear and definitive answers concerning safety/un-safety of certain GM plants?”, according to our understanding, would require spelling out the values and assumptions (regarding, for example, the sufficient evidence for safety) behind risk assessment. This would greatly contribute to building trust and solving the controversy between uncertainty and demand for safety, at least when it is accompanied by predictability in decision-making, taking responsibilities, and conferring some possibility to citizen-consumers to really affect the decision-making.
